# SARS-CoV-2 Outbreak on a Spanish Mink Farm: Epidemiological, Molecular, and Pathological Studies

**DOI:** 10.3389/fvets.2021.805004

**Published:** 2022-01-21

**Authors:** Juan José Badiola, Alicia Otero, Eloisa Sevilla, Belén Marín, Mirta García Martínez, Marina Betancor, Diego Sola, Sonia Pérez Lázaro, Jenny Lozada, Carolina Velez, Álvaro Chiner-Oms, Iñaki Comas, Irving Cancino-Muñoz, Eva Monleón, Marta Monzón, Cristina Acín, Rosa Bolea, Bernardino Moreno

**Affiliations:** ^1^Centro de Encefalopatías y Enfermedades Transmisibles Emergentes, Facultad de Veterinaria, Universidad de Zaragoza, Instituto Agroalimentario de Aragón (IA2), Instituto de Investigación Sanitaria de Aragón (IISA), Zaragoza, Spain; ^2^Facultad de Ciencias Veterinarias, Universidad Nacional de La Pampa, General Pico, Argentina; ^3^Instituto de Biomedicina de Valencia-Consejo Superior de Investigaciones Científicas (IBV-CSIC), Valencia, Spain; ^4^Instituto de Biomedicina de Valencia-Consejo Superior de Investigaciones Cientìficas (IBV-CSIC), CIBER in Epidemiology and Public Health, Valencia, Spain

**Keywords:** antibodies, mink, polymerase chain reaction, SARS-CoV-2, sequence, Spain

## Abstract

Farmed minks have been reported to be highly susceptible to severe acute respiratory syndrome coronavirus 2 (SARS-CoV-2) and may represent a risk to humans. In this study, we describe the first outbreak of SARS-CoV-2 occurred on a mink farm in Spain, between June and July 2020, involving 92,700 animals. The outbreak started shortly after some farm workers became seropositive for SARS-CoV-2. Minks showed no clinical signs compatible with SARS-CoV-2 infection throughout the outbreak. Samples from 98 minks were collected for histopathological, serological, and molecular studies. Twenty out of 98 (20.4%) minks were positive by RT-qPCR and 82 out 92 (89%) seroconverted. This finding may reflect a rapid spread of the virus at the farm with most of the animals overcoming the infection. Additionally, SARS-CoV-2 was detected by RT-qPCR in 30% of brain samples from positive minks. Sequencing analysis showed that the mink sequences were not closely related with the other mink SARS-CoV-2 sequences available, and that this mink outbreak has its probable origin in one of the genetic variants that were prevalent in Spain during the first COVID-19 epidemic wave. Histological studies revealed bronchointerstitial pneumonia in some animals. Immunostaining of viral nucleocapsid was also observed in nasal turbinate tissue. Farmed minks could therefore constitute an important SARS-CoV-2 reservoir, contributing to virus spread among minks and humans. Consequently, continuous surveillance of mink farms is needed.

## Introduction

Severe acute respiratory syndrome coronavirus 2 (SARS-CoV-2) is the causative agent of coronavirus disease 2019 (COVID-19), a declared pandemic that has affected more than 150 million people worldwide (1).

Natural SARS-CoV-2 infections have been documented in domestic dogs, felids, and mustelids. Nevertheless, to date only farmed minks (*Neovison vison*) have been shown not only to be susceptible to a natural infection, but also to develop severe illness and transmit SARS-CoV-2 to other minks and humans (2).

Europe is the global leader in fur production, with ~2,750 mink farms (3). Farming conditions could contribute to virus spread among minks, as they are kept in large groups and housed with bedding that generates dust (4). Since SARS-CoV-2 infection in farmed minks was first identified in The Netherlands in April 2020, it has now been reported on mink farms from other countries, including the USA, Denmark, Spain (the present case), France, Italy, Sweden, Canada, Greece, Lithuania, and Poland (5).

Outbreaks on mink farms are characterized by respiratory signs and increased mortality (6), although no clinical signs have been reported in some cases (7). Furthermore, virus evolution in farmed mink has led to the emergence of SARS-CoV-2 mink-associated variants, and transmission to humans and further community spread have already been described (8, 9).

The rapid virus dissemination among minks and mink-to-human transmission of SARS-CoV-2 make farmed minks a potential virus reservoir in which mutations may occur, complicating thus control of this pandemic (10).

Here, we report the first description of an outbreak of SARS-CoV-2 occurred in a mink farm in Spain in May-July 2020.

## Materials and Methods

### Outbreak Description

During the second half of May, a human outbreak of COVID-19 was detected in the province of Teruel, Autonomous Community of Aragon (Spain). Some of these positive individuals were working on a mink farm located in this province. This farm had a mink population of 19,500 adult animals (15,600 females and 3,900 males) and 73,200 kits, and had its own feed factory and slaughter facility. This farm is the only mink farm located in the Autonomous Community of Aragon. No other mink farms in its vicinity and no epidemiological links with other farms have been detected.

Since the connection between infected humans and the mink farm was established, hygienic and biosecurity measures were immediately reinforced. This farm was supervised by official veterinary authorities, and movement restrictions were applied for minks, animal products, and workers.

To investigate the presence of the virus on the farm, four consecutive official samplings of the minks were scheduled between 27th May and 7th July. After virus detection on the farm, official authorities ordered culling of all farmed minks, as well as official cleaning and disinfection of the facilities.

Most animals involved in the present study were clinically normal, randomly selected among minks culled between 17th and 21st July 2020; however, our research had already started in May with the study of individual animals dead due to causes non-related to respiratory pathology.

### Mink Necropsies and Sample Collection

After euthanasia of the minks, necropsies were performed in a Biosecurity Level 3 necropsy room at the Center for Encephalopathies and Transmissible Emerging Diseases of the University of Zaragoza, Spain. A total of 98 minks were necropsied and sampled.

Samples were collected using a set of sterile tweezers, scissors, and scalpel for each animal. In addition, surfaces were thoroughly decontaminated between necropsies using a solution of 10% bleach in water (v/v) to avoid cross contamination.

From each mink, duplicate samples were collected from the following organs: nasal turbinates, trachea, lung, liver, spleen, brain, kidney, heart, small intestine, and mesenteric lymph node. One of these samples was placed in 10% buffered formalin for histopathological study. The other sample was collected in RNAlater solution (Invitrogen), kept at 4°C for 24 h and then frozen at −80°C for RNA extraction and subsequent reverse transcription quantitative PCR (RT-qPCR) analysis.

To perform the antibody test against SARS-CoV-2, a blood clot was taken directly from the heart of the animal and placed in a sterile Eppendorf tube, which was immediately frozen at −80°C.

### Histopathological Study

Samples collected in formalin were embedded in paraffin wax and cut (4 μm thick) using a microtome. Samples collected during the necropsy were stained using hematoxylin-eosin for the analysis of histological lesions.

Several lung samples were subjected to immunohistochemical analysis for the detection of T lymphocytes. Briefly, sections were deparaffinizated and rehydrated and then subjected to hydrated autoclaving for 20 min at 96°C in a Tris-EDTA (pH 8) solution. After cooling, sections were treated with a peroxidase blocking solution (Dako) for 10 min followed by 10% goat serum for 30 min to reduce non-specific binding. Immunodetection was performed using an anti-CD3 antibody (Ready to use solution, Dako, catalog number: IR503) at room temperature (RT) for 1 h followed by an enzyme-conjugated anti-rabbit Envision polymer (Dako) for 1 h at RT, and diaminobenzidine (DAB, Dako) as the chromogen. Sections were counterstained using hematoxylin and mounted with DPX.

Additionally, an immunohistochemical analysis for viral detection was performed in PCR-positive samples. In this case, the primary monoclonal antibody mouse anti SARS-CoV NucleoProtein (Sino Biological, 40143-MM08) was used at a dilution of 1:2,000 for 1 h at RT. Then, an enzyme-conjugated anti-mouse Envision polymer (Dako) was added for 1 h at RT, and diaminobenzidine (DAB, Dako) as the chromogen. Sections were counterstained using hematoxylin. Mink nasal turbinate tissue with a low Ct value was used as a positive control and PCR-negative tissue was included as a negative control.

### Virus Detection by RT-qPCR

A total of 20 mg from each type of tissue, from each sampled mink was subjected to RNA extraction using a RNeasy Plus Mini Kit purification kit (Qiagen), following the instructions from the manufacturer.

Nasal turbinate samples were especially considered for PCR analyses because previous studies in minks have demonstrated that this type of tissue has the highest viral load (11). Additionally, a selection of 10 minks showing the highest viral load in nasal turbinate tissue was further analyzed for other types of tissue, that is trachea, lung, brain, small intestine, and mesenteric lymph node.

A kit for the specific detection of SARS-CoV-2 with internal control (CoVID-19 dtec-RT-qPCR Test, Genetic PCR SolutionsTM) was used in this case. According to the manufacturer's recommendation, 5 μl of previously extracted RNA were added by duplicate to a final volume of 20 μl for the RT-qPCR reaction. In addition, positive and negative controls were included in the microplates. A standard curve dilution series was also prepared to obtain sample quantification.

The RT-qPCR reaction was carried out in a 7500 Fast Real-Time PCR System thermal cycler (Applied Biosystems). In this case, fluorogenic signals corresponding to FAM, for SARS-CoV-2, and HEX, for the internal amplification control, were measured.

Finally, the obtained results were analyzed using 7500 Fast Software v2.0.6, thus obtaining the cycle threshold (Ct) values, and corresponding viral concentration, for each of the tested samples.

### Sequencing of SARS-CoV-2 Isolates

In order to obtain the whole sequence of the virus, we selected five samples with Ct values ≤ 30 of the diagnostic RT-qPCR result. These isolates were sequenced and analyzed following an approach described previously (12). Briefly, RNA was retro-transcribed into cDNA and SARS-CoV-2 complete genome amplification was conducted in two multiplex PCR, accordingly to the protocol developed by the ARTIC network (13) and using the V3 multiplex primers schemes (14). Genomic libraries were constructed with the Nextera DNA Flex Sample Preparation kit (Illumina Inc., San Diego, CA) according to the manufacturer's protocol, with 5 cycles for PCR indexing. The MiSeq platform (Illumina) was used for whole genome sequencing (2 × 150 cycles paired-end run).

The sequences obtained went through a bioinformatic pipeline based on IVAR (15), which is open source and can be accessed at https://gitlab.com/fisabio-ngs/sars-cov2-mapping. The pipeline goes through the following steps: (1) filtering of the fastq files using fastp v 0.20.1 5 (16) (arguments: –cut tail, –cut-window-size, –cut-mean-quality, -max_len1,-max_len2); (2) mapping and variant calling using bwa and IVAR v 1.2 [variant calling cut-offs: minimum quality for SNP calling = 20, minimum frequency to call a SNP = 0.05, minimum depth for calling a SNP = 20, consensus construction cut-offs: minimum quality for consensus calling = 20, minimum frequency to consider fixed a SNP = 0.8, minimum position depth = 30 (ambiguous base otherwise)].

### Alignment and Phylogeny Construction

We have generated two different alignments files, one including all the genomic sequences available in GISAID by 15/09/2021 having *N. vison* and *Mustela lutreola* as host (*n* = 936); and another with all the sequences generated by the SeqCOVID-Spain consortium by 15/09/2021 (*n* = 21,585). In both files, the 5 mink sequences were included and aligned against the SARS-CoV-2 reference genome (17) using MAFFT (18). Specific positions that have been reported to be problematic for phylogenetic reconstruction (19) were masked, following the procedure described by Lanfear (20), using the mask_alignment.sh script.

Finally, two maximum-likelihood (ML) phylogenies were build using IQTREE (21), with the GTR model and based on the complete masked genome alignment. Both phylogenies were rooted to the SARS-CoV-2 sequence obtained in Wuhan on 24/12/2019 (GISAID ID: EPI_ISL_402123).

### Detection of Antibodies by ELISA

ID Screen SARS-CoV-2 Double Antigen Multi-species ELISA, for the detection in serum, plasma, or whole blood of antibodies targeting the nucleocapsid of SARS-CoV-2, was performed following the manufacturer's protocol. Briefly, blood clots were disrupted using intense vortexing and centrifuged at 2,000 rpm for 10 min. A total volume of 25 μl of sample, as well as positive and negative controls, were transferred to the ELISA microplate, diluted 1:2 with the provided sample buffer and incubated at 37°C for 45 min. The ELISA microplate is coated with recombinant SARS-CoV-2 nucleocapsid protein antigen. Wells were washed 5 times with the provided wash buffer (300 μl per well and wash) and the conjugate was added and incubated at room temperature for 30 min. After a second wash step, the substrate solution was added, and the microplate was incubated in darkness at room temperature for 20 min. After addition of the stop solution, the absorbance of the wells was immediately measured at 450 nm in a TECAN Sunrise spectrophotometer. Absorbance data were analyzed as recommended by the ELISA kit manufacturer and the S/P ratio (Sample to Positive ratio) was calculated for each sample.

## Results

The first positive mink was detected on 21st of May in an adult female which had not died of a respiratory pathology. Further studies carried out on 97 culled minks showed that 19 additional animals were positive by RT-qPCR in nasal turbinates [20.4% total prevalence (95% CI: 12.4–28.4)]. Only five animals showed Ct values lower than 30, indicating that most of the animals had low viral loads in positive nasal turbinate samples (Supplementary Table 1). Interestingly, among those positive minks selected for further analysis, 3 out of 10 [30% prevalence (95% CI: 1.6–58.4)] showed PCR positivity in the brain, with Ct values ranging from 28.54 to 36.21 (Table 1). The rest of tested tissues, that is trachea, lung, small intestine, and mesenteric lymph node, from these selected individuals were considered negative (not shown).

**Table 1:** SARS-CoV-2 RT-qPCR results obtained from nasal turbinate and brain samples of mink from a Spanish farm.

**ID**	**Sample**	**SARS-CoV-2 RT-qPCR**			
		**Result**	Ct^*^	Copies/μl^**^	**Copies/mg tissue**
V13	Nasal turbinate	+	19.46	24,633.55	61,583.88
V14	Nasal turbinate	+	29.38	61.36	153.40
V15	Nasal turbinate	+	30.79	23.30	58.25
V22	Nasal turbinate	+	31.97	10.64	26.60
V24	Nasal turbinate	+	33.91	3.27	8.18
V41	Nasal turbinate	+	28.08	121.05	302.63
V51	Nasal turbinate	+	22.72	3,216.89	8,042.23
V63	Nasal turbinate	+	31.18	17.25	43.13
V67	Nasal turbinate	+	30.54	27.20	68
V74	Nasal turbinate	+	28.61	85.56	213.90
V13	Brain	+	28.54	64.41	161.03
V14	Brain	+	36.21	0.29	0.73
V15	Brain	–	na	na	na
V22	Brain	–	na	na	na
V24	Brain	–	na	na	na
V41	Brain	–	na	na	na
V51	Brain	+	31.52	7.68	19.2
V63	Brain	–	na	na	na
V67	Brain	–	na	na	na
V74	Brain	–	na	na	na

*na, not applicable*.

* *Ct values were calculated as the mean of Ct values obtained by duplicate*.

** *Sample quantification was obtained based on a standard curve performed using a serial dilution of recombinant SARS-CoV-2 RNA provided in the RT-qPCR kit used in the study*.

**Figure 1:**
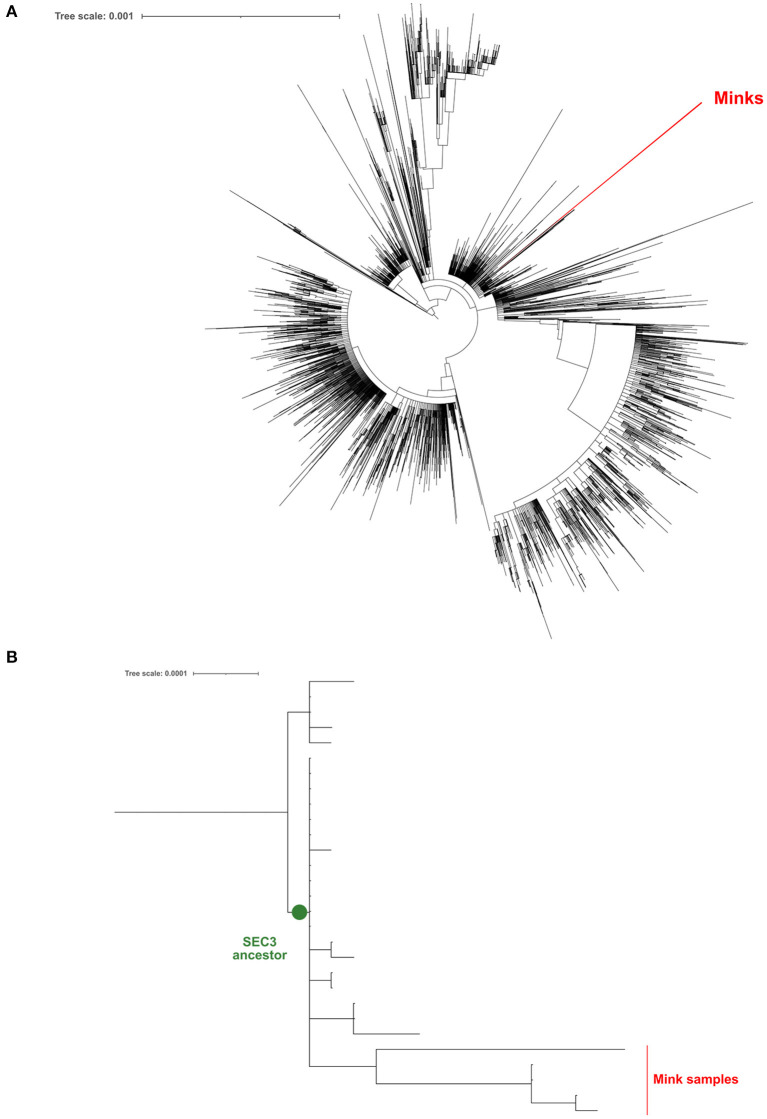
Maximum-likelihood phylogeny constructed using the genomic sequences obtained from mink samples and all the complete sequences processed by the SeqCOVID consortium at 15/09/2021. **(A)** Phylogeny tree; **(B)** A zoomed view of the phylogeny, focusing on the SEC3 clade.

Five positive samples were successfully sequenced (>90% genomic coverage, >1400X median depth) and these sequences were deposited in GISAID [Accession ID EPI_ISL_6885298 (V13), EPI_ISL_6885299 (V14), EPI_ISL_6885300 (V41), EPI_ISL_6885301 (V51), and EPI_ISL_6885302 (V74)]. All samples belong to the B.1 PANGO lineage (22). In order to discover whether these viral samples appeared due to a human contact or were the result of a zoonotic infection, we have generated a ML phylogeny with these 5 sequences and 21,585 additional genomes sequenced from clinical samples of COVID-19 Spanish patients in the frame of the SeqCOVID-SPAIN consortium. In this phylogeny we observed that the 5 mink sequences form a monophyletic group and that they derived from the previously identified Spanish Epidemic Clade 3 (SEC 3) [(12); Figure 1]. SEC3 was one of the genetic variants that dominated the first COVID-19 epidemic wave in Spain.

In addition, we generated a phylogeny including all the sequences downloaded from GISAID (*n* = 936) that were sampled from minks. Again, our 5 sequences form a unique clade away from the closest mink sequence (Figure 1).

We have found different non-synonymous mutations in the S gene for all 5 samples in comparison to the Wuhan reference strain. Three mutations are common to all samples (N501T, D614G, and I993I), while 1 is found only in V41 (N1125T) and 4 are found only in V51 (D795H, Y144F, F485V, and F140del) (Supplementary Table 2). Most of these mutations are “new” as they have not been found in the ancestral SEC3 node (except for D614G). We have also checked the abundance of these mutations in the minks' samples that we have downloaded from GISAID. Mutation D614G is present in all of them. N501T is also abundant (124 sequences) and Y144F is found only in 1 sequence. Rest of the mutations (I993I, N1125T, F485V, and F140del) are unique to the Spanish minks, although it is common to find other amino acid substitutions or deletions in these codons.

Serological studies showed that 82 out of 92 (89%) minks tested positive for SARS-CoV-2 specific antibodies, confirming the circulation of the virus on the farm. Antibodies were detected in all of the RT-qPCR positive minks, with the exception of one (V92).

**Figure 2:**
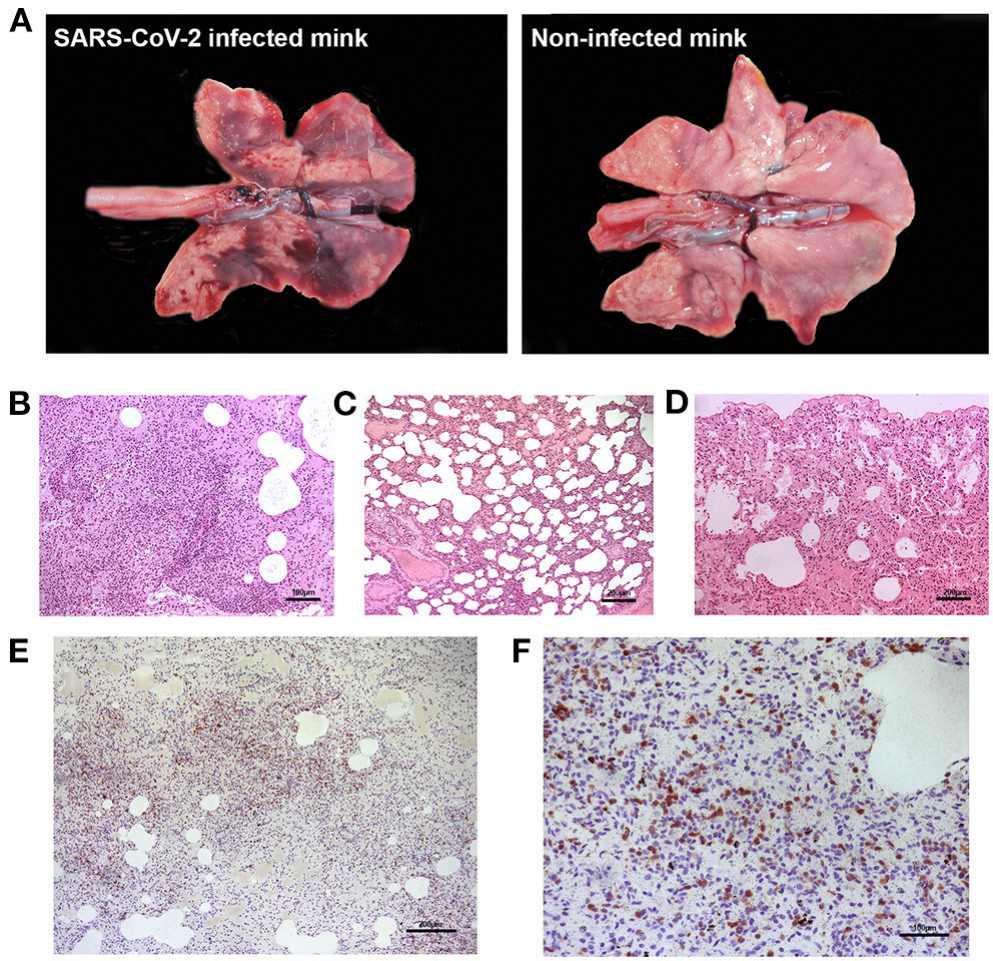
Gross and microscopic pulmonary lesions in minks infected with SARS-CoV-2. **(A)** Gross lesions are characterized by irregular dark areas of consolidation in the lungs of infected minks (left). Right panel shows the lungs from a non-infected, healthy mink not presenting dark consolidated areas. **(B)** Perivascular inflammatory infiltrates and bronchointerstitial pneumonia with alveolitis are observed in one positive mink (H-E, x100). **(C)** Thickened alveolar walls by inflammatory infiltrates in one positive mink (H-E, x50). **(D)** Normal histological features in the lung of a mink negative for SARS-CoV-2. **(E)** Marked proliferation of T lymphocytes in the lung interstitium of one positive mink, confirmed by immunohistochemistry using an anti-CD3 antibody (x50). **(F)** Magnified capture of **(E)** showing immunopositive T lymphocytes (x100).

**Figure 3:**
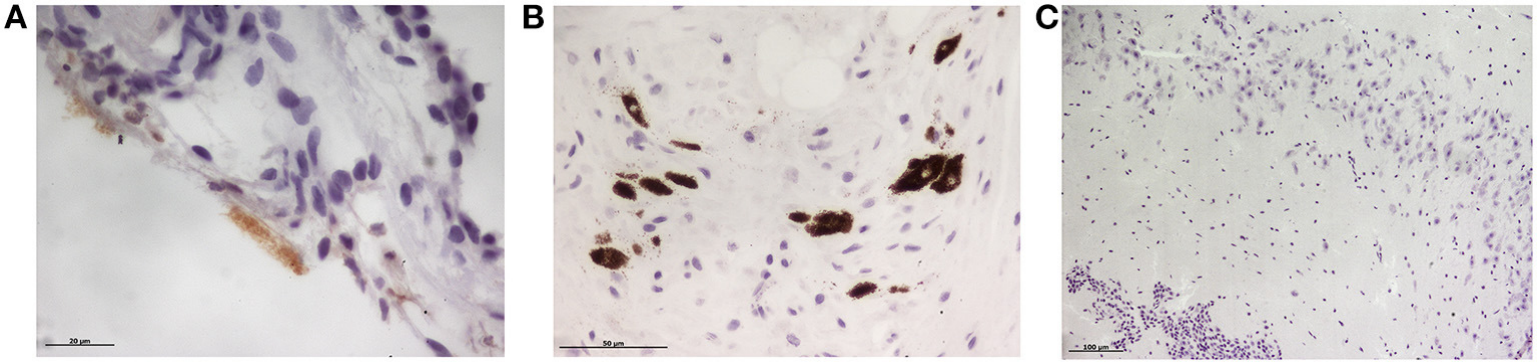
Immunohistochemical detection of SARS-CoV-2 nucleoprotein in mink tissues. **(A)** Detection of SARS-CoV-2 N protein in the olfactory epithelium (x400). **(B)** Immunodetection for N protein was also detected in olfactory neurons (x400). **(C)** No immunopositivity was detected in brain samples although viral RNA was amplified by RT-PCR in three animals (x100).

None of the minks included in this study showed clinical signs of respiratory disease. Since the first positive case in this farm was detected (May 2020) to the date of culling, no significant increase in mortality was observed (~2% of mortality was considered as normal). Macroscopically, lung lesions were observed in a few minks and were characterized by irregular areas of consolidation (Figure 2); however, microscopic lesions were found in more animals, especially in SARS-CoV-2 PCR positive minks and in minks PCR-negative but positive for SARS-CoV-2 antibodies. Histopathological findings revealed a mild to moderate bronchointerstitial pneumonia characterized by pulmonary congestion and edema, thickening of alveolar septa by mononuclear cells and the presence of peribronchiolar and perivascular infiltrates. Immunostaining with an anti-CD3 antibody demonstrated the presence of numerous T lymphocytes both within the pulmonary interstitium and around airways and blood vessels (Figure 2). Mild chronic rhinitis was found in a few minks. No lesions were observed in the trachea of any animal. In addition, immunohistochemical analysis for viral detection revealed cells positive for SARS-CoV-2 nucleoprotein within nasal turbinate samples. Within these samples, immunostaining was observed in nasal epithelial cells and cells whose morphology was compatible with olfactory neurons. However, no positive result was observed in brain samples subjected to immunohistochemistry (Figure 3). In addition, no histological lesions were observed in brain tissues from mink in which positivity was detected in this tissue by RT-qPCR.

It is also worth mentioning that, during the outbreak, mortality, and feed intake parameters were considered within normal values. In addition, no clinical signs compatible with SARS-CoV-2 infection were observed among the farmed minks.

## Discussion

This study describes the first outbreak of SARS-CoV-2 on a mink farm in Spain. This farm had no epidemiological links with other farms and transmission most probably occurred by human contact, as several farm workers tested positive before the outbreak started on the farm. To test this, our phylogenetic analyses showed that the mink sequences were not closely related with the other mink sequences available. In fact, this outbreak has its origin in one of the genetic variants that were previously identified as prevalent in Spain during the first COVID-19 epidemic wave (12).

Although increased mortality, decreased feed intake and respiratory symptoms have been described as the most frequent clinical signs related to SARS-CoV-2 infection in minks (6, 7), none of these signs were observed throughout the present outbreak. In fact, it has also been estimated that minks from one third of farms may remain asymptomatic (7).

A large proportion (86.67%) of animals resulted positive for SARS-CoV-2 by RT-qPCR on 7th July 2020, under the official analyses (23). However, our study demonstrated a 20% of positive minks by RT-qPCR. This lower prevalence could be explained by the fact that minks selected for the study were sampled after culling, that is, on 17–21th July. During this period (2 weeks approximately), most animals could have overcome the viral infection and become PCR-negative when analyzed for this study.

We analyzed a small cohort of animals from this farm. These animals were collected from the farm during one of the worst waves of the COVID-19 pandemic in Spain. The analysis of these minks was carried out when the availability of RT-PCR and antibody tests was highly limited since they were destined to human diagnosis. Additionally, official authorities did not allow us to manipulate more animals at that moment due to health risks and unawareness of the COVID-19 disease in mink. However, we consider that we analyzed a representative sample of animals from the farm.

It is worthy to note that 30% of brain tissue samples tested were also PCR-positive. Nevertheless, the rest of the analyzed tissues were considered negative, suggesting that SARS-CoV-2 had not disseminated to other organs in minks. These results could be explained by a viral invasion at the neural–mucosal interface in olfactory mucosa, followed by transport along the olfactory tract (24). Unlike a recent study in which SARS-CoV-2 was detected by RT-PCR in different organs including brain from two naturally infected minks (25), in our case other organs such as trachea or lung remained negative. However, those minks had died from typical SARS-CoV-2 infection respiratory symptoms, while individuals from this study had no clinical symptoms. This could also explain why no histopathological lesions were found in the brains of our PCR-positive minks, whereas Chaintoutis et al. found fibrinous meningitis in these affected animals. However, although we used sterile equipment during the necropsies, we cannot rule out the possibility of a potential contamination of these brain samples considering that those 3 minks were highly positive by PCR in nasal turbinates, which are in close proximity to the brain.

Serological studies revealed that almost 90% of the minks showed SARS-CoV-2 specific antibodies, which supports the rapid spread of the virus at the farm. These results agree with previously reported serological studies, which obtained similar seroprevalences (11, 26). In addition, the proportion of seropositive animals is very close to the prevalence described at the beginning of the outbreak (7th July 2020: 86.67%), as previously mentioned.

None of the analyzed animals showed signs of respiratory disease, which explains the lack of macroscopic pulmonary lesions in most animals. However, interstitial pneumonia characterized by thickened alveolar walls and perivascular/bronchiolar lymphoid infiltrates were found microscopically in some minks. Similar lung lesions were found in a study in which minks were intranasally exposed to SARS-CoV-2, although the lesions described here were more severe since these minks developed clinical disease (27). These pulmonary lesions, however, can be caused by other respiratory viruses in mink, such as influenza or adenovirus. However, periodic analyses for respiratory diseases are performed in this farm, and the animals were free of these diseases shortly before the culling was performed.

In line with PCR results, immunohistochemical analysis for SARS-CoV-2 nucleoprotein detection showed positivity in nasal turbinate tissue. However, viral immunostaining was not observed in brain samples, likely due to the lower sensitivity of this laboratory technique.

Virus evolution in farmed mink has led to the emergence of SARS-CoV-2 mink-associated variants, and transmission to humans and further community spread have already been described (8, 9, 26). Particularly, mink-derived SARS-CoV-2 sequences from the Netherlands and Denmark have been shown to contain multiple substitutions in the S protein receptor binding domain (RBD) that increase the mean binding energy, suggestive of potential adaptation of the S protein to the mink angiotensin-converting enzyme 2 (ACE2) receptor (28). Consequently, the emergence of novel mink-associated variants might have direct impacts on diagnostic tests, treatments, and developed vaccine candidates (29).

In this work the mink samples can be traced back to a dominant variant in human samples during the first wave in Spain. However, as observed elsewhere, it also harbors a range of mutations that are convergently found in other mink samples (https://www.biorxiv.org/content/10.1101/2020.11.16.384743v1). Thus, those mutations are likely associated with adaptation from humans to minks. Novel mutations are also found in the sequences, suggesting that there are several adaptive options that could successfully drive adaptation to minks. Among mutations observed in mink SARS-CoV-2 spike sequences, Y453F and D614G mutations may confer a better affinity for the mink ACE2 and/or decreasing sensitivity to the neutralizing immune response (30). In this study, only the latter was detected in all sequenced genomes. However, it has been described that the D614G mutation, although being more transmissible, is not associated with an increase in the disease severity (31). This could explain the results that we observed in the mink farm. Apart from that, N501T mutation, which is also found in all our mink sequences, has been described recently to have higher affinity to human ACE2 receptor (32). However, its relationship with the severity of the disease is still to be determined. Therefore, we consider that the consequences of the mink SARS-CoV-2 mutations observed in this study should be further investigated regarding affinity for ACE2 and viral pathogenesis.

In conclusion, farmed minks could constitute a relevant reservoir of the virus since the presence of asymptomatic individuals, as occurred in the Spanish outbreak, could difficult the detection of SARS-CoV-2 infection, and thus contribute to virus spread among minks and humans. For this reason, continuous surveillance of mink farms is therefore needed. However, the relevance of minks in the epidemiology of SARS-CoV-2 in humans is yet to be established.

## Data Availability Statement

The datasets presented in this study can be found in online repositories. The name of the repository and accession numbers can be found in the article/Supplementary Material.

## Ethics Statement

Ethical review and approval was not required for the animal study because samples used in the present study were collected from minks euthanized by official authorities following EU regulations for animal welfare.

## Author Contributions

BMa and BMo collected the minks used in the study. AO, ES, BMa, MG, MB, DS, JL, SP, and BMo performed the mink necropsies. AO, ES, MG, MB, DS, JL, SP, and CV performed the RT-PCR and the ELISA techniques. AO, ES, MG, MB, DS, and SP analyzed the RT-PCR and ELISA data. AO and ES performed the hematoxylin-eosin and immunohistochemistry techniques. JB and BMo analyzed the histopathological results and obtained the funding for the study. ÁC-O, IC, and IC-M performed and analyzed the sequence analysis, and the alignment and phylogeny construction. JB, AO, ES, ÁC-O, IC, IC-M, and BMo wrote the original draft. JB, EM, MM, CA, RB, and BMo curated the data, revised and edited the draft, and generated the final version of the manuscript. All authors contributed to the article and approved the submitted version.

## Funding

This study was funded by Banco Santander S.A. and University of Zaragoza, through the project titled Estudio de la importancia de los animales de compañía como reservorios del SARS-CoV-2 y en la epidemiología de la enfermedad COVID-19. In addition, genome sequencing was funded by Fondo COVID COV20/00140.

## Conflict of Interest

This study received funding from Banco Santander S.A. and University of Zaragoza. The funder was not involved in the study design, collection, analysis, interpretation of data, the writing of this article, or the decision to submit it for publication. The authors declare that the research was conducted in the absence of any commercial or financial relationships that could be construed as a potential conflict of interest.

## Publisher's Note

All claims expressed in this article are solely those of the authors and do not necessarily represent those of their affiliated organizations, or those of the publisher, the editors and the reviewers. Any product that may be evaluated in this article, or claim that may be made by its manufacturer, is not guaranteed or endorsed by the publisher.
