# Supplementary material for: SARS-CoV-2 Outbreak on a Spanish Mink Farm: Epidemiological, Molecular, and Pathological Studies
Source: Front Vet Sci. 2022 Jan 21;8:805004. doi: 10.3389/fvets.2021.805004 (PMC8814420; doi:10.3389/fvets.2021.805004)
Supplement: Supplementary file 1 [file Table_1.pdf]

Supplementary table 1: Detection of SARS-CoV-2 by RT-qPCR in nasal turbinate samples and serology results from minks collected from a Spanish farm

	SARS-CoV-2 RT-qPCR				
**ID**	**Result**	**Ct***	**Copies/µl****	**Copies/mg tissue**	**SARS-CoV-2 ELISA**
V3	-	na	na	na	ND
**V4**	**+**	**17.97**	**139,075.03**	**347,687.58**	ND
V5	-	na	na	na	ND
**V6**	**+**	**36.55**	**1.26**	**3.15**	ND
V7	-	na	na	na	ND
V8	-	na	na	na	ND
**V13**	**+**	**19.46**	**24,633.55**	**61,583.88**	**+**
**V14**	**+**	**29.38**	**61.36**	**153.40**	**+**
**V15**	**+**	**30.79**	**23.30**	**58.25**	**+**
V16	-	na	na	na	+
**V17**	**+**	**35.10**	**1.45**	**3.63**	**+**
**V18**	**+**	**37.95**	**0.24**	**0.60**	**+**
V19	-	na	na	na	+
**V20**	**+**	**35.47**	**1.15**	**2.58**	**+**
**V21**	**+**	**39.51**	**0.04**	**0.10**	**+**
**V22**	**+**	**31.97**	**10.64**	**26.60**	**+**
**V23**	**+**	**34.22**	**2.52**	**6.30**	**+**
**V24**	**+**	**33.91**	**3.27**	**8.18**	**+**
V25	-	na	na	na	-
**V26**	**+**	**38.48**	**0.17**	**0.43**	**+**
V27	-	na	na	na	+
V28	-	na	na	na	+
V29	-	na	na	na	+
V30	-	na	na	na	-
V31	-	na	na	na	+
V32	-	na	na	na	-
V33	-	na	na	na	+
V34	-	na	na	na	+
V35	-	na	na	na	-
V36	-	na	na	na	+
V37	-	na	na	na	+
V38	-	na	na	na	+
V39	-	na	na	na	+
V40	-	na	na	na	+

**V41**	**+**	**28.08**	**121.05**	**302.63**	**+**
V42	-	na	na	na	+
V43	-	na	na	na	+
V44	-	na	na	na	+
V45	-	na	na	na	+
V46	-	na	na	na	+
V47	-	na	na	na	+
V48	-	na	na	na	+
V49	-	na	na	na	+
V50	-	na	na	na	+
**V51**	**+**	**22.72**	**3,216.89**	**8,042.23**	**+**
V52	-	na	na	na	+
V53	-	na	na	na	+
V54	-	na	na	na	-
V55	-	na	na	na	+
V56	-	na	na	na	+
V57	-	na	na	na	+
V58	-	na	na	na	+
V59	-	na	na	na	+
V60	-	na	na	na	+
V61	-	na	na	na	+
V62	-	na	na	na	+
**V63**	**+**	**31.18**	**17.25**	**43.13**	**+**
V64	-	na	na	na	+
V65	-	na	na	na	+
V66	-	na	na	na	+
**V67**	**+**	**30.54**	**27.20**	**68**	**+**
V68	-	na	na	na	+
V69	-	na	na	na	+
V70	-	na	na	na	+
V71	-	na	na	na	+
V72	-	na	na	na	+
V73	-	na	na	na	+
**V74**	**+**	**28.61**	**85.56**	**213.90**	**+**
V75	-	na	na	na	+
V76	-	na	na	na	+

V77	-	na	na	na	+
V78	-	na	na	na	-
V79	-	na	na	na	+
V80	-	na	na	na	+
V81	-	na	na	na	-
V82	-	na	na	na	+
V83	-	na	na	na	+
V84	-	na	na	na	+
**V85**	**+**	**34.37**	**5.99**	**14.98**	**+**
V86	-	na	na	na	-
V87	-	na	na	na	+
V88	-	na	na	na	+
V89	-	na	na	na	+
V90	-	na	na	na	+
V91	-	na	na	na	+
**V92**	**+**	**34.61**	**5.08**	**12.70**	**-**
V93	-	na	na	na	+
V94	-	na	na	na	+
V95	-	na	na	na	+
V96	-	na	na	na	+
V97	-	na	na	na	-
V98	-	na	na	na	+
V99	-	na	na	na	+
V100	-	na	na	na	+
V101	-	na	na	na	+
V102	-	na	na	na	+
V103	-	na	na	na	+
V104	-	na	na	na	+

na: not applicable; ND: Not done

*Ct values were calculated as the mean of Ct values obtained by duplicate.

**Sample quantification was obtained based on a standard curve, which was calculated using a dilution series provided in the RT-qPCR kit used in the study.
