# Supplementary material for: SARS-CoV-2 Outbreak on a Spanish Mink Farm: Epidemiological, Molecular, and Pathological Studies
Source: Front Vet Sci. 2022 Jan 21;8:805004. doi: 10.3389/fvets.2021.805004 (PMC8814420; doi:10.3389/fvets.2021.805004)
Supplement: Supplementary file 2 [file Table_2.docx]

| **ID mink** | **Mutations** |
| --- | --- |
| V13^a^, V14^b^, V41^c^, V51^d^, V74^e^ | N501T D614G I993I |
| V13, V14, V41, V74 | G652G (syn) |
| V41 | N1125T |
| V51 | D795H Y144F F485V F140del |

**Supplementary table 2: Nonsynonymous mutations detected in the S gene of Spanish minks in comparison to the Wuhan reference strain**

GISAID accession ID: ^a^ EPI_ISL_6885298, ^b^EPI_ISL_6885299, ^c^EPI_ISL_6885300, ^d^EPI_ISL_6885301, ^e^EPI_ISL_6885302
